# 3-(2-Fluoro­phen­yl)-6-(phenoxy­meth­yl)-1,2,4-triazolo[3,4-*b*][1,3,4]thia­diazole

**DOI:** 10.1107/S1600536808003917

**Published:** 2008-03-12

**Authors:** Melanie Holm, Dieter Schollmeyer, Stefan Laufer

**Affiliations:** a Institute of Pharmacy, Department of Pharmaceutical and Medicinal Chemistry, Eberhard-Karls-University Tübingen, Auf der Morgenstelle 8, 72076 Tübingen, Germany; b Department of Organic Chemistry, Johannes Gutenberg-University Mainz, Duesbergweg 10-14, D-55099 Mainz, Germany

## Abstract

The crystal structure of the title compound, C_16_H_11_FN_4_OS, was synthesized in the course of our studies on 1,2,4-triazolo[3,4-*b*][1,3,4]thia­diazo­les as inhibitors of p38 mitogen-activated protein kinase (MAPK). The three-dimensional data obtained were used to generate a three-dimensional pharmacophore model for *in silico* database screening. The dihedral angles between the central heterocylic system and the fluoro­phenyl and phenyl rings are 20.21 (3) and 5.43 (1)°, respectively; the dihedral angle between the two benzene rings is 15.80 (4)°.

## Related literature

Protein kinases (PK) are favoured targets for the development of new drugs (Hopkins & Groon, 2002 ▶) because the reversible protein-phospho­rylation by PK is an important control mechanism in the signal pathways of a cell (Laufer *et al.*, 2005 ▶). The [1,2,4]triazolo[3,4-*b*][1,3,4]thia­diazole nucleus is associated with diverse biological activities (Malhotra *et al*., 2003 ▶). For the preparation of the title compound, see: Invidiata *et al*. (1997 ▶); Malhotra *et al.* (2003 ▶).
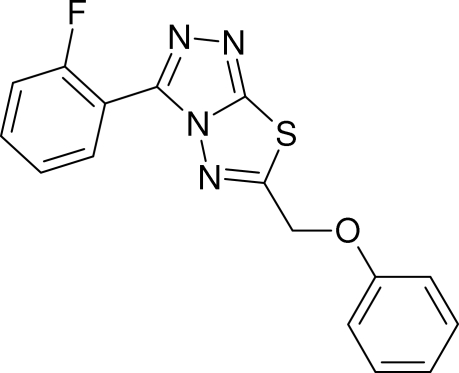


## Experimental

### 

#### Crystal data

C_16_H_11_FN_4_OS
*M* *_r_* = 326.35
Monoclinic, 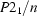

*a* = 10.8551 (6) Å
*b* = 12.1899 (3) Å
*c* = 11.6667 (6) Å
β = 110.857 (5)°
*V* = 1442.61 (11) Å^3^
*Z* = 4
Cu *K*α radiation
μ = 2.19 mm^−1^
*T* = 193 (2) K
0.58 × 0.51 × 0.26 mm

#### Data collection

Enraf–Nonius CAD-4 diffractometer
Absorption correction: ψ scan (*CORINC*; Dräger & Gattow, 1971 ▶) *T*_min_ = 0.61, *T*_max_ = 0.99 (expected range = 0.348–0.565)
2883 measured reflections
2736 independent reflections
2583 reflections with *I* > 2σ(*I*)
*R*_int_ = 0.037
3 standard reflections frequency: 60 min intensity decay: 4%

#### Refinement

*R*[*F* ^2^ > 2σ(*F* ^2^)] = 0.048
*wR*(*F* ^2^) = 0.135
*S* = 1.06
2736 reflections
209 parameters
H-atom parameters constrained
Δρ_max_ = 0.43 e Å^−3^
Δρ_min_ = −0.43 e Å^−3^

### 

Data collection: *CAD-4 Software* (Enraf–Nonius, 1989 ▶); cell refinement: *CAD-4 Software*; data reduction: *CORINC* (Dräger & Gattow, 1971 ▶); program(s) used to solve structure: *SIR92* (Altomare *et al.*, 1994 ▶); program(s) used to refine structure: *SHELXL97* (Sheldrick, 2008 ▶); molecular graphics: *PLATON* (Spek, 2003 ▶); software used to prepare material for publication: *SHELXL97*.

## Supplementary Material

Crystal structure: contains datablocks I, global. DOI: 10.1107/S1600536808003917/bt2678sup1.cif

Structure factors: contains datablocks I. DOI: 10.1107/S1600536808003917/bt2678Isup2.hkl

Additional supplementary materials:  crystallographic information; 3D view; checkCIF report

## Figures and Tables

**Fig. 1.**
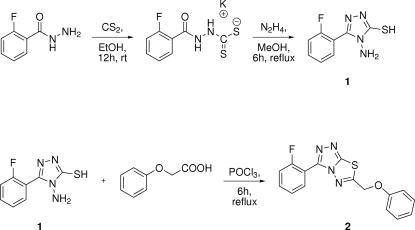
Synthesis of compounds 1 and 2.

**Fig. 2.**
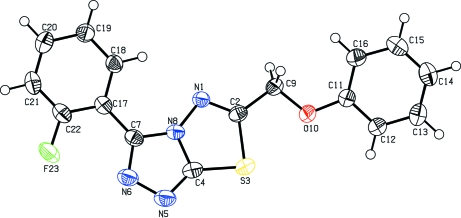
Perspective view of 2. Displacement ellipsoids are drawn at the 50% probability level. H atoms are depicted as circles of arbitrary size.

## supplementary crystallographic information

### Comment

Continued interest in development of small molecule inhibitors of p38 mitogen-activated protein (MAP) kinase is based on the central role of this enzyme in inflammatory cell signalling. Activation of p38 leads to an increase production of pro-inflammatory cytokines such as TNF-alpha and IL-1beta. Many different diseases have their seeds in an overactive immune response. Prominent examples like psoriasis, rheumatoid arthritis and inflammatory bowel disease turn it into a still prominent target for antiinflammatory drug discovery. 3-(2-chlorophenyl)-6-((4-methoxyphenoxy)methyl)-[1,2,4]triazolo[3,4-*b*] [1,3,4]thiadiazole was identified as potential hit in a virtual screening and the [1,2,4]triazolo[3,4-*b*][1,3,4]thiadiazole core therefore chosen as starting point for a medicinal chemistry program. To gain more information about structure-activity relationship, a series of compounds were synthesized and tested.

The synthesis of 1 (Figure 1) was started from the substituted hydrazide, which was treated with carbon disulfide. Cyclization followed by reaction with hydrazine. The final product 2 was synthesized by reaction of 1 with 2-phenoxyacetic acid in presence of phosphorus oxychloride.

Of special interest was the proposed binding mode of disubstituted compound and a crystal structure of compound 2 was prepared (Figure 2).

### Experimental

The synthesis of 3-(2-fluorophenyl)-6-(phenoxymethyl)-[1,2,4]triazolo[3,4-*b*][1,3,4]thiadiazole was started from 2-fluorobenzohydrazide. Carbon disulfide (55.0 mmol) was added slowly to a solution of the hydrazide (36.0 mmol) in absolute ethanol (70 ml) containing potassium hydroxid (55.0 mmol). The resulting mixture was stirred over night at room temperature, then cooled and diluted with ether (100 ml). Potassium 2-(2-fluorobenzoyl)hydrazinecarbodithioate precipitated and was collected by filtration, washed with diethyl ether and dried.

A mixture containing potassium dithiocarbazinate (16.0 mmol) suspended in water (2 ml) and hydrazine hydrate (99%, 32.0 mmol) was heated under gentle reflux for 2 h. It was cooled to room temperature and diluted with water (80 ml) and acidified with concentrated hydrochloric acid. Thick white solid mass separated out. It was collected by filtration, washed with water and recrystallized from ethanol to get 4-amino-5-(2-fluorophenyl)-4*H*-1,2,4-triazole-3-thiol 1. (Invidiata *et al.*, 1997)

For the preparation of the title compound, a mixture of 1 (5.0 mmol), 2-phenoxyacetic acid (1.0 mmol) and phosphorus oxychloride (10 ml) was refluxed for 6 h, cooled to room temperature and poured onto crushed ice. The solid product separated out and was collected by filtration, washed with aqueous NaOH solution (20 ml, 2 *M*) and then with water, dried and recrystallized from ethanol 2. (Malhotra *et al.*, 2003)

Crystals of 2 for X-ray analysis precipitated slowly as brown platelets from ethanol at room temperature.

### Refinement

Hydrogen atoms were placed at calculated positions with C—H=0.95A% (aromatic) or 0.99 Å (*sp*^3^ C-atom). All H atoms were refined with isotropic displacement parameters set at 1.2 times of the *U*_eq_ of the parent atom.

### Crystal data

**Table:**

C_16_H_11_FN_4_OS	*F*_000_ = 672
*M**_r_* = 326.35	*D*_x_ = 1.503 Mg m^−^^3^
Monoclinic, *P*2_1_/*n*	Cu *K*α radiation λ = 1.54178 Å
Hall symbol: -P 2yn	Cell parameters from 25 reflections
*a* = 10.8551 (6) Å	θ = 65–70º
*b* = 12.1899 (3) Å	µ = 2.19 mm^−^^1^
*c* = 11.6667 (6) Å	*T* = 193 (2) K
β = 110.857 (5)º	Plate, light brown
*V* = 1442.61 (11) Å^3^	0.58 × 0.51 × 0.26 mm
*Z* = 4	

### Data collection

**Table:**

Enraf–Nonius CAD-4 diffractometer	*R*_int_ = 0.037
Monochromator: graphite	θ_max_ = 69.9º
*T* = 193(2) K	θ_min_ = 4.8º
ω/2θ scans	*h* = 0→13
Absorption correction: ψ scan(CORINC; Dräger & Gattow, 1971)	*k* = 0→14
*T*_min_ = 0.61, *T*_max_ = 0.99	*l* = −14→13
2883 measured reflections	3 standard reflections
2736 independent reflections	every 60 min
2583 reflections with *I* > 2s(*I*)	intensity decay: 4%

### Refinement

**Table:**

Refinement on *F*^2^	Hydrogen site location: inferred from neighbouring sites
Least-squares matrix: full	H-atom parameters constrained
*R*[*F*^2^ > 2σ(*F*^2^)] = 0.048	*w* = 1/[σ^2^(*F*_o_^2^) + (0.0847*P*)^2^ + 0.6884*P*] where *P* = (*F*_o_^2^ + 2*F*_c_^2^)/3
*wR*(*F*^2^) = 0.135	(Δ/σ)_max_ < 0.001
*S* = 1.06	Δρ_max_ = 0.43 e Å^−^^3^
2736 reflections	Δρ_min_ = −0.43 e Å^−^^3^
209 parameters	Extinction correction: SHELXL97 (Sheldrick, 2008), Fc^*^=kFc[1+0.001xFc^2^λ^3^/sin(2θ)]^-1/4^
Primary atom site location: structure-invariant direct methods	Extinction coefficient: 0.0071 (8)
Secondary atom site location: difference Fourier map	

### Special details

**Table:**

Geometry. All e.s.d.'s (except the e.s.d. in the dihedral angle between two l.s. planes) are estimated using the full covariance matrix. The cell e.s.d.'s are taken into account individually in the estimation of e.s.d.'s in distances, angles and torsion angles; correlations between e.s.d.'s in cell parameters are only used when they are defined by crystal symmetry. An approximate (isotropic) treatment of cell e.s.d.'s is used for estimating e.s.d.'s involving l.s. planes.
Refinement. Refinement of *F*^2^ against ALL reflections. The weighted *R*-factor *wR* and goodness of fit *S* are based on *F*^2^, conventional *R*-factors *R* are based on *F*, with *F* set to zero for negative *F*^2^. The threshold expression of *F*^2^ > σ(*F*^2^) is used only for calculating *R*-factors(gt) *etc*. and is not relevant to the choice of reflections for refinement. *R*-factors based on *F*^2^ are statistically about twice as large as those based on *F*, and *R*- factors based on ALL data will be even larger.

### Fractional atomic coordinates and isotropic or equivalent isotropic displacement parameters (Å^2^)

**Table:**

	*x*	*y*	*z*	*U*_iso_*/*U*_eq_	
N1	0.65749 (15)	0.29912 (12)	0.27951 (14)	0.0311 (4)	
C2	0.62295 (17)	0.25592 (16)	0.17147 (17)	0.0324 (4)	
S3	0.63057 (5)	0.11269 (4)	0.16086 (4)	0.0375 (2)	
C4	0.68622 (19)	0.11015 (15)	0.31907 (19)	0.0358 (4)	
N5	0.72177 (18)	0.03789 (14)	0.40767 (17)	0.0445 (4)	
N6	0.75709 (18)	0.09854 (14)	0.51539 (17)	0.0426 (4)	
C7	0.74119 (17)	0.20439 (15)	0.48900 (18)	0.0331 (4)	
N8	0.69477 (14)	0.21442 (12)	0.36353 (14)	0.0306 (4)	
C9	0.58296 (19)	0.32345 (16)	0.05735 (17)	0.0358 (4)	
H9A	0.5190	0.3806	0.0593	0.043*	
H9B	0.6607	0.3596	0.0484	0.043*	
O10	0.52441 (15)	0.24906 (12)	−0.04087 (12)	0.0433 (4)	
C11	0.50482 (17)	0.28429 (16)	−0.15846 (17)	0.0326 (4)	
C12	0.45545 (19)	0.20490 (17)	−0.24815 (18)	0.0376 (4)	
H12	0.4366	0.1333	−0.2266	0.045*	
C13	0.4337 (2)	0.23046 (19)	−0.36915 (19)	0.0436 (5)	
H13	0.4012	0.1759	−0.4308	0.052*	
C14	0.4591 (2)	0.3350 (2)	−0.40103 (19)	0.0451 (5)	
H14	0.4442	0.3524	−0.4843	0.054*	
C15	0.5060 (2)	0.41365 (19)	−0.3114 (2)	0.0439 (5)	
H15	0.5220	0.4858	−0.3336	0.053*	
C16	0.5303 (2)	0.38932 (16)	−0.1887 (2)	0.0378 (5)	
H16	0.5637	0.4437	−0.1271	0.045*	
C17	0.77445 (17)	0.29576 (16)	0.57653 (17)	0.0323 (4)	
C18	0.76314 (19)	0.40498 (16)	0.53692 (18)	0.0345 (4)	
H18	0.7312	0.4204	0.4515	0.041*	
C19	0.79764 (19)	0.49076 (17)	0.61989 (18)	0.0390 (4)	
H19	0.7895	0.5642	0.5909	0.047*	
C20	0.8439 (2)	0.47064 (19)	0.74481 (19)	0.0426 (5)	
H20	0.8664	0.5297	0.8016	0.051*	
C21	0.8569 (2)	0.3633 (2)	0.78588 (19)	0.0451 (5)	
H21	0.8895	0.3480	0.8713	0.054*	
C22	0.8224 (2)	0.27895 (18)	0.70268 (19)	0.0399 (5)	
F23	0.83678 (15)	0.17577 (12)	0.74746 (12)	0.0608 (4)	

### Atomic displacement parameters (Å^2^)

**Table:**

	*U*^11^	*U*^22^	*U*^33^	*U*^12^	*U*^13^	*U*^23^
N1	0.0311 (7)	0.0273 (8)	0.0325 (8)	0.0000 (6)	0.0082 (6)	0.0011 (6)
C2	0.0281 (8)	0.0299 (9)	0.0381 (10)	−0.0027 (7)	0.0105 (7)	−0.0040 (7)
S3	0.0402 (3)	0.0294 (3)	0.0430 (3)	−0.00168 (17)	0.0147 (2)	−0.00653 (18)
C4	0.0330 (10)	0.0276 (10)	0.0457 (11)	−0.0003 (7)	0.0126 (8)	−0.0033 (8)
N5	0.0482 (10)	0.0293 (9)	0.0522 (10)	0.0041 (7)	0.0134 (8)	0.0039 (7)
N6	0.0453 (10)	0.0317 (9)	0.0464 (10)	0.0027 (7)	0.0111 (8)	0.0066 (7)
C7	0.0283 (8)	0.0320 (10)	0.0375 (10)	0.0007 (7)	0.0099 (7)	0.0059 (7)
N8	0.0279 (7)	0.0244 (7)	0.0375 (8)	0.0009 (6)	0.0092 (6)	0.0011 (6)
C9	0.0390 (10)	0.0318 (10)	0.0325 (10)	−0.0051 (7)	0.0075 (8)	−0.0044 (7)
O10	0.0606 (9)	0.0355 (8)	0.0316 (7)	−0.0147 (6)	0.0137 (6)	−0.0050 (6)
C11	0.0304 (9)	0.0348 (10)	0.0322 (9)	0.0007 (7)	0.0107 (7)	−0.0011 (7)
C12	0.0398 (10)	0.0348 (10)	0.0375 (10)	−0.0024 (8)	0.0129 (8)	−0.0026 (8)
C13	0.0416 (10)	0.0532 (13)	0.0341 (10)	0.0000 (9)	0.0112 (8)	−0.0062 (9)
C14	0.0370 (10)	0.0612 (14)	0.0369 (11)	0.0034 (10)	0.0129 (8)	0.0081 (10)
C15	0.0377 (10)	0.0433 (11)	0.0495 (12)	0.0011 (9)	0.0141 (9)	0.0132 (9)
C16	0.0358 (10)	0.0340 (11)	0.0425 (11)	−0.0018 (7)	0.0125 (8)	−0.0018 (8)
C17	0.0262 (8)	0.0365 (10)	0.0334 (9)	0.0009 (7)	0.0097 (7)	0.0038 (7)
C18	0.0345 (9)	0.0356 (10)	0.0314 (9)	0.0036 (8)	0.0094 (7)	0.0030 (7)
C19	0.0412 (10)	0.0364 (10)	0.0378 (10)	0.0054 (8)	0.0119 (8)	0.0006 (8)
C20	0.0404 (10)	0.0500 (13)	0.0377 (10)	0.0005 (9)	0.0144 (8)	−0.0083 (9)
C21	0.0468 (11)	0.0600 (14)	0.0293 (10)	−0.0021 (10)	0.0144 (8)	0.0045 (9)
C22	0.0384 (10)	0.0428 (11)	0.0384 (10)	−0.0014 (8)	0.0136 (8)	0.0110 (9)
F23	0.0822 (10)	0.0482 (8)	0.0443 (7)	−0.0076 (7)	0.0128 (7)	0.0186 (6)

### Geometric parameters (Å, °)

**Table:**

N1—C2	1.292 (2)	C13—C14	1.382 (3)
N1—N8	1.381 (2)	C13—H13	0.9500
C2—C9	1.492 (3)	C14—C15	1.376 (3)
C2—S3	1.7544 (19)	C14—H14	0.9500
S3—C4	1.726 (2)	C15—C16	1.392 (3)
C4—N5	1.307 (3)	C15—H15	0.9500
C4—N8	1.363 (2)	C16—H16	0.9500
N5—N6	1.389 (3)	C17—C22	1.391 (3)
N6—C7	1.324 (2)	C17—C18	1.400 (3)
C7—N8	1.373 (2)	C18—C19	1.383 (3)
C7—C17	1.467 (3)	C18—H18	0.9500
C9—O10	1.422 (2)	C19—C20	1.384 (3)
C9—H9A	0.9900	C19—H19	0.9500
C9—H9B	0.9900	C20—C21	1.384 (3)
O10—C11	1.380 (2)	C20—H20	0.9500
C11—C16	1.382 (3)	C21—C22	1.371 (3)
C11—C12	1.385 (3)	C21—H21	0.9500
C12—C13	1.381 (3)	C22—F23	1.349 (2)
C12—H12	0.9500		
			
C2—N1—N8	107.31 (15)	C12—C13—H13	119.8
N1—C2—C9	122.45 (17)	C14—C13—H13	119.8
N1—C2—S3	118.02 (15)	C15—C14—C13	119.5 (2)
C9—C2—S3	119.48 (13)	C15—C14—H14	120.2
C4—S3—C2	87.12 (9)	C13—C14—H14	120.2
N5—C4—N8	111.49 (18)	C14—C15—C16	121.1 (2)
N5—C4—S3	138.59 (15)	C14—C15—H15	119.4
N8—C4—S3	109.92 (14)	C16—C15—H15	119.4
C4—N5—N6	105.39 (16)	C11—C16—C15	118.55 (19)
C7—N6—N5	109.71 (16)	C11—C16—H16	120.7
N6—C7—N8	107.62 (17)	C15—C16—H16	120.7
N6—C7—C17	126.80 (18)	C22—C17—C18	116.43 (19)
N8—C7—C17	125.48 (17)	C22—C17—C7	122.11 (18)
C4—N8—C7	105.78 (16)	C18—C17—C7	121.42 (17)
C4—N8—N1	117.63 (15)	C19—C18—C17	121.19 (18)
C7—N8—N1	136.59 (15)	C19—C18—H18	119.4
O10—C9—C2	105.77 (15)	C17—C18—H18	119.4
O10—C9—H9A	110.6	C18—C19—C20	120.62 (19)
C2—C9—H9A	110.6	C18—C19—H19	119.7
O10—C9—H9B	110.6	C20—C19—H19	119.7
C2—C9—H9B	110.6	C21—C20—C19	119.1 (2)
H9A—C9—H9B	108.7	C21—C20—H20	120.4
C11—O10—C9	117.99 (15)	C19—C20—H20	120.4
O10—C11—C16	124.62 (17)	C22—C21—C20	119.70 (19)
O10—C11—C12	114.51 (17)	C22—C21—H21	120.2
C16—C11—C12	120.87 (18)	C20—C21—H21	120.2
C13—C12—C11	119.58 (19)	F23—C22—C21	117.38 (19)
C13—C12—H12	120.2	F23—C22—C17	119.7 (2)
C11—C12—H12	120.2	C21—C22—C17	122.9 (2)
C12—C13—C14	120.3 (2)		
			
N8—N1—C2—C9	−176.82 (15)	C9—O10—C11—C12	175.96 (17)
N8—N1—C2—S3	0.50 (19)	O10—C11—C12—C13	−179.06 (17)
N1—C2—S3—C4	0.04 (15)	C16—C11—C12—C13	1.2 (3)
C9—C2—S3—C4	177.45 (15)	C11—C12—C13—C14	−1.0 (3)
C2—S3—C4—N5	179.1 (2)	C12—C13—C14—C15	−0.1 (3)
C2—S3—C4—N8	−0.59 (14)	C13—C14—C15—C16	1.0 (3)
N8—C4—N5—N6	−0.9 (2)	O10—C11—C16—C15	179.97 (18)
S3—C4—N5—N6	179.41 (19)	C12—C11—C16—C15	−0.4 (3)
C4—N5—N6—C7	0.4 (2)	C14—C15—C16—C11	−0.8 (3)
N5—N6—C7—N8	0.2 (2)	N6—C7—C17—C22	−3.4 (3)
N5—N6—C7—C17	−176.25 (17)	N8—C7—C17—C22	−179.30 (17)
N5—C4—N8—C7	1.0 (2)	N6—C7—C17—C18	174.60 (19)
S3—C4—N8—C7	−179.19 (12)	N8—C7—C17—C18	−1.2 (3)
N5—C4—N8—N1	−178.73 (15)	C22—C17—C18—C19	−0.3 (3)
S3—C4—N8—N1	1.1 (2)	C7—C17—C18—C19	−178.43 (17)
N6—C7—N8—C4	−0.7 (2)	C17—C18—C19—C20	−0.3 (3)
C17—C7—N8—C4	175.79 (17)	C18—C19—C20—C21	0.9 (3)
N6—C7—N8—N1	178.96 (18)	C19—C20—C21—C22	−0.8 (3)
C17—C7—N8—N1	−4.5 (3)	C20—C21—C22—F23	−179.92 (18)
C2—N1—N8—C4	−1.0 (2)	C20—C21—C22—C17	0.2 (3)
C2—N1—N8—C7	179.34 (19)	C18—C17—C22—F23	−179.53 (17)
N1—C2—C9—O10	−167.07 (16)	C7—C17—C22—F23	−1.4 (3)
S3—C2—C9—O10	15.6 (2)	C18—C17—C22—C21	0.3 (3)
C2—C9—O10—C11	−165.85 (15)	C7—C17—C22—C21	178.45 (19)
C9—O10—C11—C16	−4.3 (3)		
